# Exploration of Stock Portfolio Investment Construction Using Deep Learning Neural Network

**DOI:** 10.1155/2022/7957097

**Published:** 2022-05-10

**Authors:** Zizheng Xie, Yi Wang

**Affiliations:** Economics and Management School, Wuhan University, Wuhan 430072, China

## Abstract

To study the intelligent and efficient stock portfolio in China's financial market, based on the relevant theories such as deep learning (DL) neural network (NN) and stock portfolio, this study selects 111 stable stocks from the constituent stocks of the China Security Index (CSI) 300 from January 1, 2018, to December 31, 2021, as the research samples. Then, it analyzes these research samples and imports the relevant data of 111 stocks into the DL NN model. The corresponding prediction results of stock prices are obtained. Finally, the stock portfolio model based on DL NN is compared with the data results of the Shanghai Stock Exchange (SSE) 50 Index and CSI 500 Index. The results show that the closing prices of the selected 111 stocks are relatively stable and fluctuate up and down around the horizontal axis, and the positive and negative returns are relatively balanced, roughly between −5% and 5%. There is a phenomenon of fluctuation aggregation to a certain extent. Comparing the prediction results of different models reveals that the prediction results of model *c* are closest to the actual stock price trend. Comparing the relevant returns of the proposed stock portfolio with other stocks uncovers that the annualized return of the stock portfolio based on the DL NN model is 47.44%. The sharp ratio is 1.52, the maximum pullback is 18.15%, the monthly excess return is 3.11%, and the information ratio is 0.82. Compared with other indexes, the proposed stock portfolio shows the best results. Therefore, the proposal of the stock portfolio based on DL NN provides a theoretical basis for the development of the financial field in the future.

## 1. Introduction

The financial sector plays a vital role in the sustainable development of the national economy. With the fast-forwarding socioeconomic level and the gradual opening of the financial market, more and more opportunities have been brought to investors [1, 2]. Therefore, how to maximize the return on investment (ROI) is one of the key issues that every investor or enterprise pays attention to for a long time. A stock portfolio is a collection of stocks, bonds, and financial derivatives held by investors or financial institutions, aiming to spread the risk. A portfolio can be seen as a combination at several levels. The first level is the combination of risk assets and risk-free assets due to the dual needs of safety and profitability. The second level is to consider how to combine risky assets. The stock portfolio based on deep learning (DL) neural network (NN) shows the advantage of a relatively high ROI rate in the financial market. Accordingly, this study will deeply analyze the stock portfolio using DL NN [3].

De la Torre-Torres et al. put forward the capital asset pricing model, which held that the relationship between the expected return of assets and asset risk was linear. The capital asset pricing model was simple and clear but had obvious limitations. The model's assumptions were difficult to realize in real life [4]. Mussafi and Ismail analyzed theoretical formulations and solutions used in portfolio selections, discussing the construction of portfolio models and the corresponding extension problems. Years of research and development have accumulated a large amount of research data in the portfolio investment field. It is less feasible to process and calculate the data by relying on traditional data analysis methods. Therefore, experts and scholars have gradually started to use DL to conduct investment portfolio research [5]. Ping and Hao used a deep reinforcement learning (DRL)-based model to solve the dynamic portfolio optimization problem. Dynamic portfolio optimization allocated funds to a series of assets in sequence according to the investor's return and risk status during continuous trading cycles. They simulated trading with realistic constraints using real financial market historical data and demonstrated that the proposed model achieved higher returns than previous benchmark trading strategies and model-free DRL models [6]. Mensi et al. constructed a portfolio optimization model based on DRL and improved the traditional mean-variance model. They found that the new model performed well in risk prediction and control through model backtesting, proving that the model had a certain practical value [7].

**Figure 1:**
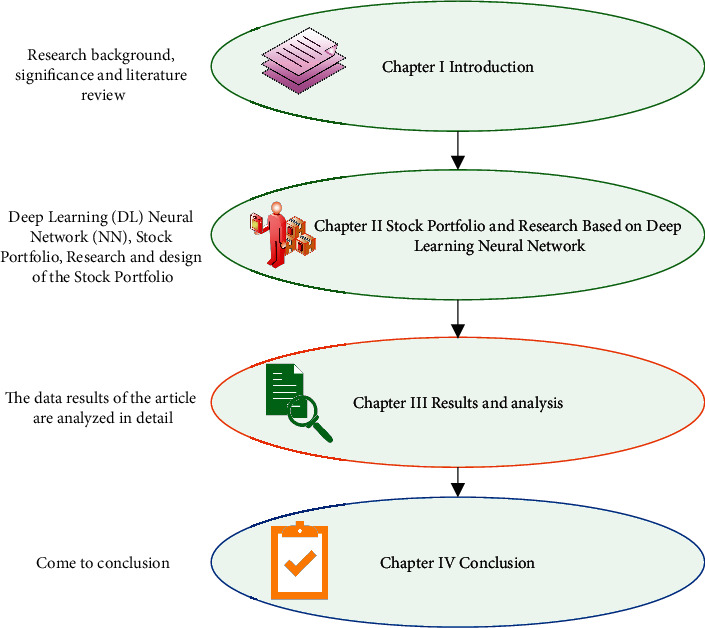
Organizational structure.

To sum up, based on DL NN, this study takes 111 stable stocks in Shanghai Stock Exchange (SSE) and Shenzhen Stock Exchange (SHZ) from January 1, 2018, to December 31, 2021, as the research object. Then, it makes an in-depth analysis and adds these stocks to the DL NN model to predict the stock price trend. Finally, the stock trends of the SSE 50 Index and Chinese Security Index (CSI) 500 are compared with them, and the result of the stock portfolio based on DL NN is the best. This study aimed to provide a methodological reference for future research on constructing stock portfolio. The organizational structure of this study is shown in Figure 1.

## 2. Stock Portfolio and Research Based on Deep Learning Neural Network

### 2.1. Deep Learning (DL) Neural Network (NN)

**Figure 2:**
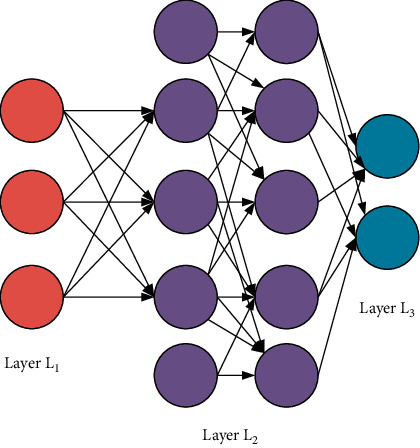
DL model.

DL stems from machine learning (ML) as a new research direction. ML studies how computers simulate or realize the learning behavior of animals to learn new knowledge or skills, rewrite the existing data structure, and then improve the program performance. Obviously, DL is strongly related to NN in ML. In turn, NN is also DL's main algorithm and implementation means. It is fair to say that DL is an improved NN algorithm [8–10]. The theoretical model of DL is shown in Figure 2.

The calculation basis of the DL algorithm is to observe the decline degree of the error function of the NN and then correct the weight and deviation of the neuron layer accordingly [11]. The specific calculation method reads the following:

(1) $$\begin{array}{c} Q_{a+1}=Q_{a}-c_{a}d_{a}. \end{array}$$

In equation (1), *Q*_*a*+1_ represents the weight and deviation of neuron layer. *Q*_*a*_ is the weight and deviation after iterative calculation. *c*_*a*_ denotes the learning speed of NN. *d*_*a*_ stands for the gradient of the error function.

The specific calculation method of the space size of the cavity post-convolution kernel reads the following:

(2) $$\begin{array}{c} A=\left(n-1\right)*b+1. \end{array}$$

In equation (2), *A* represents the size of the convolution kernel space. *n* stands for the size of the original convolution kernel. *b* is the cavity rate. The specific calculation method of the size of the picture after convolution reads the following:

(3) $$\begin{array}{c} B=\frac{\left(l-K+2P\right)}{S+1}. \end{array}$$

In equation (3), *B* represents the size of the picture after convolution. *l* is the size of the input picture. *K* means the size of the output picture. *P* denotes the filling size of the picture. *S* signifies the step size.

Nonnegative matrix factorization (NMF) is also an effective DL method, making the final product approach to the original matrix by finding two or more nonnegative matrices. It can reduce the nonlinear data dimension [12]. The calculation of norm cost function based on matrix difference reads the following:

(4) $$\begin{array}{c} \underset{P,W,Q}{\min}{\left\|Y-PWQ^{T}\right\|}_{F}^{2}. \end{array}$$

In equation (4), *Q*^*T*^ represents the transpose of matrix *Q*. ‖.‖_*F*_ represents the norm of the matrix difference. *P*, *W*, *Q*, *Y* signify different matrices. The updated decomposition calculation method is given in the following equations:

(5) $$\begin{array}{c} P_{ij}\leftarrow P_{ij}\frac{\left(YQW^{T}\right)}{{\left(PWQ^{T}QW^{T}\right)}_{ij}}, \end{array}$$

(6) $$\begin{array}{c} W_{ij}\leftarrow W_{ij}\frac{{\left(P^{T}YQ\right)}_{ij}}{{\left(P^{T}PWQ^{T}Q\right)}_{ij}}, \end{array}$$

(7) $$\begin{array}{c} Q_{ij}\leftarrow Q_{ij}\frac{{\left(YPW\right)}_{ij}}{{\left(QW^{T}P^{T}PW\right)}_{ij}}. \end{array}$$

In equations (5)–(7), *P*_*ij*_ represents the element in row *i* by column *j* of matrix *P*. *W*_*ij*_ means the element in row *i* by column *j* of matrix *Q*. *W*^*T*^, *Q*^*T*^, *P*^*T*^ denote the transpose of different matrices, and the remaining letters share the same as the above equations.

Multitask learning (MTL), an essential part of the DL method, can solve data fragmentation in DL by looking for helpful information from relevant data [13, 14]. According to the definition of the MTL model, the specific calculation is exhibited as follows:

(8) $$\begin{array}{c} \min\sum_{m}^{M}L\left(X^{m},Y^{m},W^{m}\right)+\lambda \text{Reg}\left(W\right). \end{array}$$

In equation (8), *X*^*m*^, *Y*^*m*^, *W*^*m*^, respectively, represent the input matrices *X*, *Y*, *W* of the *m*th task. *M* is the total number of samples. Reg denotes the regularization constraints. *λ* stands for the weight controlling the regularization constraint.

Then, to ensure the research data accuracy, it is necessary to calculate the accuracy and recall, as in the following equations:

(9) $$\begin{array}{c} \text{Accuracy}=\frac{TP+TN}{P+N}, \end{array}$$

(10) $$\begin{array}{c} \text{Recall}=\frac{TP}{TP+FN}=\frac{TP}{P}. \end{array}$$

In equations (9) and (10), *TP* represents the number of positive cases correctly classified. *TN* is the number of negative cases correctly classified. *P* means *TP*+*FN*. *FN* stands for the number of incorrectly classified negative cases. Lastly, *N* indicates the total number.

Analyzing the research content of DL unveils mainly three different methods.

**Figure 3:**
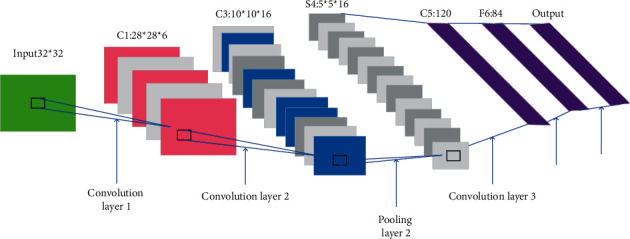
CNN model.

**Figure 4:**
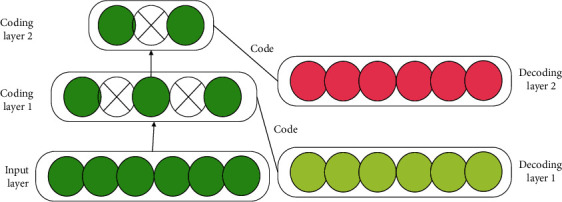
Self-coding NN model.

**Figure 5:**
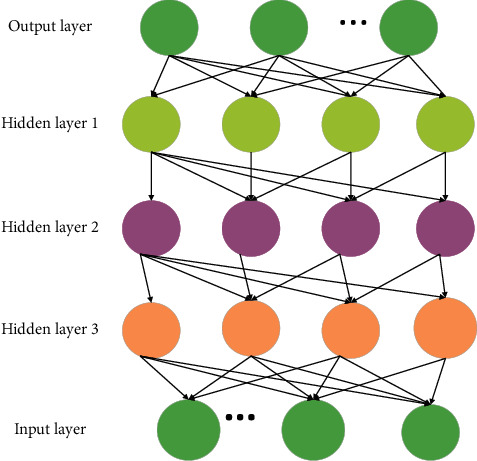
DBN model.

(1) The NN system based on convolution operation is the CNN model with convolution calculation. It calculates the feedforward NN with a depth-wise structure. CNN structures four layers: input, convolution, pooling, and full connection, and is one of the representative algorithms of DL [15, 16].  Figure 3 details the CNN architecture.
  (11) $$\begin{array}{c} R_{t}=ℑ\left(B_{t}\left[b_{t-1},x_{t}\right]+P_{f}\right), \end{array}$$
  (12) $$\begin{array}{c} Y_{t}=ℑ\left(A_{I}\cdot \left[b_{t-1},x_{t}\right]+P_{I}\right). \end{array}$$
In equations (11) and (12), *R*_*t*_, *ℑ*, *B*_*t*_, and *b*_*t*−1_ represent forget gate, hidden layer neuron, model output, and hidden layer information at *t* − 1 moment. *x*_*t*_ is the input at moment *t*. *Y*_*t*_ stands for input gate. Lastly, *P*_*f*_ and *P*_*I*_ are the activation function (AF) of forget gate and input gate, respectively.
DL is also applied in the field of stock portfolio, such as stock price prediction. The simplest method is to use the autoregressive model, as counted by the following equation:
  (13) $$\begin{array}{c} X_{t}=c+\sum_{i=1}^{P}\phi_{i}X_{t-i}+\varepsilon_{t}. \end{array}$$
In equation (13), *X*_*t*_ represents the stock price series. *X*_*t*−*i*_ is the *X*_*t*−*i*_ order lag of stock price series. *ϕ*_*i*_ indicates the weight of the lag term. *p* refers to the order. *c* stands for the constant term. *ε*_*t*_ signifies that the average is assumed to be 0. According to equation (13), linear regression can be used for verification, and its mathematical model is transformed into the following equations:
  (14) $$\begin{array}{c} X_{t}=f\left({\overset{⟶}{\Phi }}^{T}{\overset{⟶}{X}}_{t,P}+c\right), \end{array}$$
  (15) $$\begin{array}{c} {\overset{⟶}{X}}_{t,P}={\left(X_{t-1},X_{t-2},\ldots ,X_{t-P}\right)}^{T}. \end{array}$$
In equations (14) and (15), *T* represents the transposition of various equations. $\overset{⟶}{X}$ is the linear regression of stock price series. *f* means an inverse function, and the remaining letters share the same meaning as in the above equations.
(2) Self-coding NNs based on multilayer neurons include auto-encoder and sparse coding, which have recently attracted extensive attention [17, 18]. The self-encoder is a kind of artificial neural network used in semi-supervised and unsupervised learning. It can characterize and learn the input information. The self-encoder represents the learning algorithm in a general sense and is applied to dimensionality reduction and outlier detection. The self-encoder constructed with a convolution layer can be applied to computer vision problems, including image noise reduction and neural style migration, as explained in Figure 4.
(3) The deep belief network (DBN) is pretrained through multilayer self-coding NN and then combined with identification information to optimize NN's weights. The model can be used for both unsupervised learning and supervised learning [19, 20]. DBN is a probability generation model. Compared with the neural network of the traditional discrimination model, the generation model establishes a joint distribution between observation data and labels. The whole neural network can generate training data according to the maximum probability by training the weights between its neurons. DBN can identify features, classify data, and generate data. DBN algorithm is an efficient learning algorithm with a wide range of applications and strong expansibility. It can be applied to handwritten word recognition, speech recognition, and image processing in ML, as detailed in Figure 5.

The specific calculation method is presented in equations (11) and (12).

The parameter setting of the DL NN model is listed in Table 1.

### 2.2. Stock Portfolio

An investment portfolio is a collection of stocks, bonds, and financial derivatives held by investors or financial institutions to spread the risk. A portfolio can be seen as a financial asset combination at several levels. In the first level, the combination of risk assets and risk-free assets is considered due to the dual needs of safety and profitability. Risk-free assets need to be combined for safety, and risk assets need to be combined for profitability. The second level of a portfolio is to consider combining risk assets. Two asset portfolios with poor or negative correlation can be combined to yield a greater risk return than individual assets. Thus, the portfolio combination helps keep the portfolio's efficient frontier away from risks [21–23].

The relevant theories about portfolios are as follows. (1) Nonquantitative portfolio strategy: the core idea of this strategy is “do not put eggs in the same basket.” The most common classifications are fundamental analysis and technical analysis [24]. Fundamental analysis analyzes the current situation of the stock from the aspects of the economic cycle, industry, and company texture and judges its fundamental texture to make investment strategies [25]. Technical analysis refers to producing technical indicators through standard charts or constructed signal data in the capital market. Differently put, technical analysis is based on the assumption that “history can repeat itself” to predict market behavior. Then, it adopts specific strategies after the emergence of some specific index characteristics. The K-line chart analysis method is most commonly used [26]. Both methods also have some limitations. The fundamental analysis method may judge the future direction of the stock price. However, it cannot make a specific quantitative judgment, thus adding a great opportunity cost in a complex environment [27]. The technical analysis method signals the operator to judge whether and how to operate. However, it is too dependent on the subjective consciousness and experience of the operator and lacks objectivity [28]. (2) Quantitative portfolio strategy makes certain assumptions before implementation [29, 30]. First, investors are rational. Second, investors only rely on the game's mean and variance rather than external information; finally, investors are in the same single investment period [31–33]. The calculation of the mean-variance model reads the following:

(16) $$\begin{array}{c} \min\delta^{2}\left(r_{p}\right)=\sum \sum w_{i}w_{j}\mathrm{cov}\left(r_{i}r_{j}\right), \end{array}$$

(17) $$\begin{array}{c} E\left(r_{p}\right)=E\left(w_{i}r_{i}\right). \end{array}$$

In equations (16) and (17), *r*_*p*_ represents the overall PR. *r*_*i*_ indicates the income of the *i*th asset. *w*_*i*_ stands for the weight of the *i*th asset. *δ*^2^(*r*_*p*_) means the variance of the PR, namely the overall risk of the portfolio. cov(*r*_*i*_*r*_*j*_) signifies the covariance of the *i*th asset and the *j*th asset. Then, the calculation of the expected PR of a single security or portfolio reads the following:

(18) $$\begin{array}{c} {\bar{r}}_{i}=r_{f}+\beta_{i}*\left({\bar{r}}_{m}-r_{f}\right), \end{array}$$

In equation (18), *r*_*f*_ is the risk-free return rate. *β*_*i*_ is the *β* coefficient of the *i*th portfolio. $\bar{r_{m}}$ indicates the expected return rate of the overall market. ${\bar{r}}_{m}-r_{f}$ stands for market premium.

Some emergencies can also lead to the stock market's collapse, such as war and economic crisis. In particular, value at risk (VaR) can measure the changes in asset prices in extreme cases. The specific calculation reads the following:

(19) $$\begin{array}{c} {\text{Risk}}_{t}=\left(y_{t}-\mu \right)\sum^{-1}{\left(y_{t}-\mu \right)}^{\prime }\in R, \end{array}$$

In equation (19), *y*_*t*_ ∈ R represents the stock return at the current time. *μ* ∈ R means the average historical returns. ∑(*y*_*t*_ − *μ*)′ ∈ *R* indicates the covariance of historical returns. *μ* is the coefficient of price change. Then, to create a stable and profitable portfolio investment strategy, a sharp ratio (SR) needs to be used, and its specific calculation is given as follows:

(20) $$\begin{array}{c} M=\frac{{\bar{r}}_{p}-r_{f}}{\sigma_{p}}. \end{array}$$

In equation (20), *M* represents the SR. $\bar{r}$ represents the expected PR. *r*_*f*_ means the risk-free interest rate. *σ*_*p*_ refers to the portfolio standard deviation.

### 2.3. Research and Design of the Stock Portfolio

**Figure 6:**
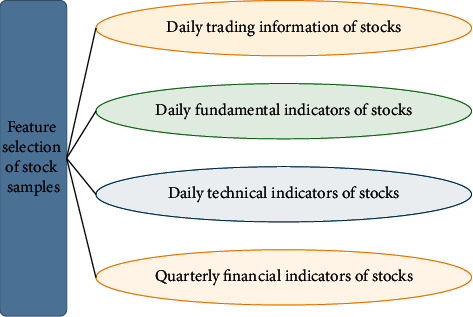
Feature selection of stock samples.

1. Sample Selection Principle. First, the selected samples must be true, accurate, and credible. Secondly, the selected relevant enterprise data should have a unified format and standard; the principle of consistency and the calculation unit and measurement method are unified. Finally, the selected data should be comparable with statistical differences. Based on the above three principles, the selected sample data have reference value and are helpful to verify the effectiveness and accuracy of the proposed model.
2. Selection of Research Objects. In this study, the constituent stocks of the CSI 300 Index after preliminary screening are used as research samples, and the time interval is all stock trading days between January 1, 2018, and December 31, 2021. However, in practice, the CSI 300 Index will be adjusted twice a year and is limited by the time range of the study. Thus, the samples are screened as follows. First, it retains the stocks that have been on the list of constituent stocks of the CSI 300 Index from 2018 to 2021. As the business and financial statement data of listed companies in the financial industry are relatively special, financial listed companies are excluded. The initial listing of stocks will cause abnormal fluctuations in the stock price, which is much greater than that of other stocks. Therefore, the stocks listed later than July 2017 are excluded. Meanwhile, the CSI 300 Index includes stocks in the ChiNext and Science and Technology Innovation Boards; these two sectors' trading rules and company characteristics are very different from the main board market. Thus, the stocks of these two plates are also excluded. After the above screening, 111 stocks are finally obtained. Then, 111 stocks are divided into three different models. The stock data of model *a* are not processed by an algorithm, the stock data of model *b* are added with other transaction information and normalized, and the stock data of model *c* are dimensionally reduced on the basis of model *b*.
3. Feature Selection of Stock samples. Stock will be affected by many factors. This study will choose from the following aspects, as unfolded in Figure 6.

## 3. Results and Analysis

### 3.1. Research on Relevant Data of Sample Stocks

**Figure 7:**
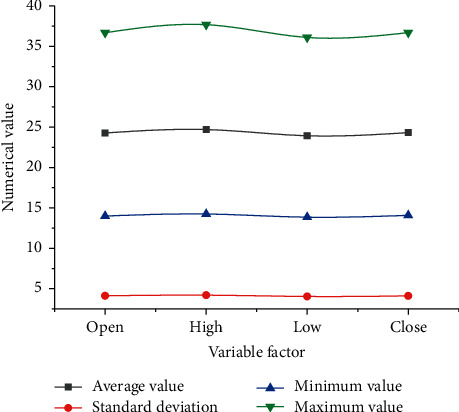
Descriptive statistics of research variables.

According to the above research design, the relevant data of the selected 111 stable constituent stocks are displayed in Figure 7.

**Figure 8:**
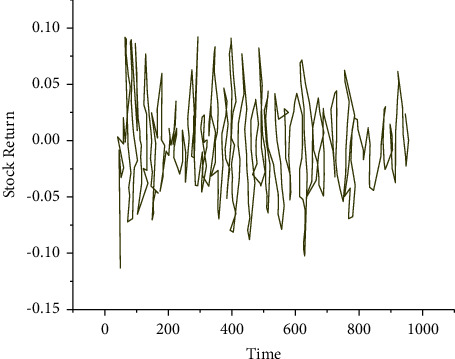
Daily closing price trend of stock.

As in Figure 7, the mean, standard deviation (SD), minimum, and maximum of the 111 stocks at the opening, the highest point, the lowest point, and the closing are (24.28, 4.12, 13.99, and 36.68), (24.7, 4.2, 14.24, and 37.68), (23.93, 4.04, 13.86, and 36.1), and (24.32, 4.11, 14.09, and 36.69), respectively. Additionally, the closing price trend of the stock is sketched in Figure 8.

From Figure 8, in the four years from January 1, 2018, to December 31, 2021, the stock's closing price is relatively stable, showing a trend of fluctuation around the horizontal axis. The positive and negative returns are relatively balanced, roughly between −5% and 5%, with certain fluctuation aggregation.

**Figure 9:**
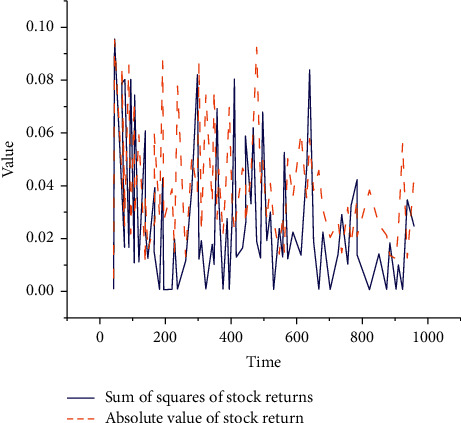
Results of SS and AV of stock returns.

Finally, the sum of squares (SS) and absolute value (AV) of the 111 stock returns are demonstrated in Figure 9.

From Figure 9, there is an obvious fluctuation aggregation in the SS and AV of the daily returns of these 111 stocks. Apparently, the stock may have an error, and the residual of the mean equation needs to be introduced to test the effect. According to the corresponding calculation and test, the final *P* value is less than 0.01. Thus, 111 stocks have passed the corresponding test.

### 3.2. Stock Portfolio Analysis Based on Deep Learning (DL) Neural Network (NN)

**Figure 10:**
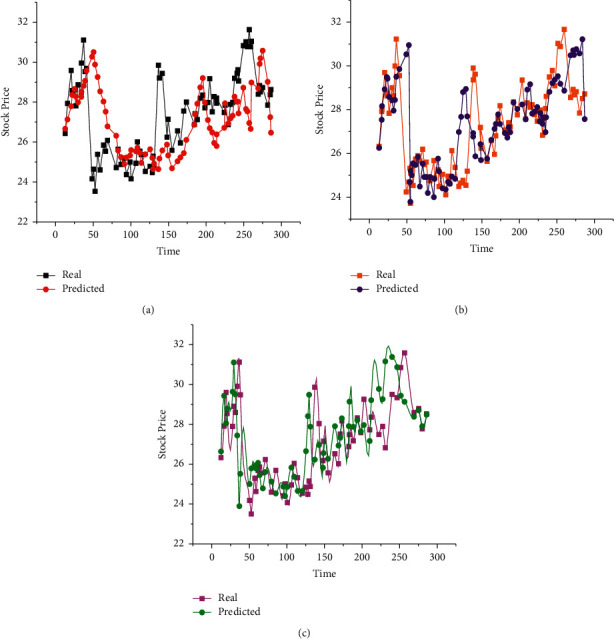
Predicted stock closing prices of different models ((a) shows the stock closing price of model *a*; (b) is the stock closing price of model *b*; and (c) illustrates the stock closing price of model *c*).

Subsequently, to study the construction of the stock portfolio from different angles, this section inputs the relevant data of 111 stocks into the DL NN model to predict stock closing price. The specific situation is counted in Figure 10.

The difference between the three models is that the stock data of model *a* are not processed by an algorithm, the stock data of model *b* are added with other transaction information and standardized, and the stock data of model *c* are dimensionally reduced on the basis of model *b*. From Figure 10, the closing price of stocks is predicted by model *a*, model *b*, and model *c*, respectively. The comparison reveals the fitness of different models' predicted and actual stock closing prices. From low to high are models *a* to *b* and *c*. Presumably, other transaction information is added to model *b*, and model *b* is standardized. Therefore, its accuracy is better than model *a*. Additionally, model *c* pretreats the standardized model *b* through dimensionality reduction. Therefore, it performs best among the three. Moreover, with the increase in input variables in the DL NN model, the final prediction results have been improved.

**Figure 11:**
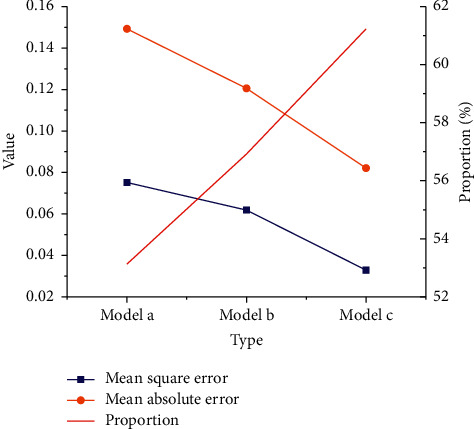
Evaluation of closing price prediction of three models.

Then, mean square error (MSE) and mean absolute error (MAE) are calculated according to the predicted stock closing price.  Figure 11 compares the evaluation indexes of the three different models.

**Figure 12:**
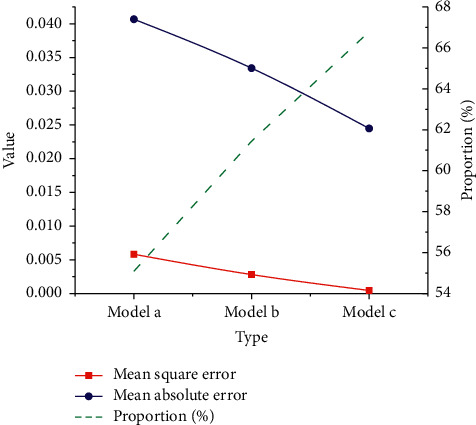
Prediction results of stock return based on the DL NN model.

As in Figure 11, the MSE, MAE, and accuracy of model a are about 0.075, about 0.149, and 53.13%. Those indexes of model *b* are about 0.062, about 0.121, and about 56.92%. Lastly, in model *c*, MSE, MAE, and accuracy are about 0.033, about 0.082, and 61.23%, respectively. Apparently, the MSE and MAE decrease slowly with the increasing model input, while the accuracy has increased from 53.13% to 61.23%. Thereupon, Figure 12 manifests the prediction results under the DL NN model.

As in Figure 12, the MSE, MAE, and accuracy of model *a* are about 0.0058, about 0.0406, and 55.09%. Those indexes of model *b* are about 0.0028, about 0.0334, and about 61.44%. Lastly, for model *c*, those indexes are about 0.0005, about 0.0245, and 66.79%, respectively. Evidently, similar to the closing price prediction results, as model input increases, the MSE and MAE decrease slowly, but the accuracy has increased from 55.09% to 66.79%.

**Figure 13:**
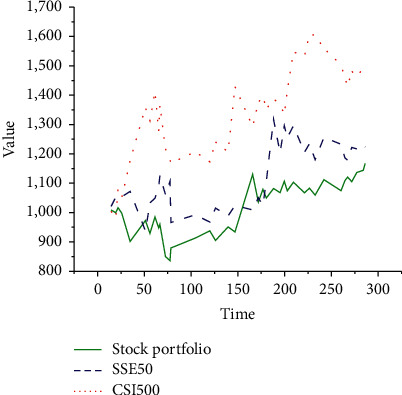
Comparison of SSE 50 Index and CSI 500 Index with DL NN-predicted stock portfolio trend.

In conclusion, after the DL NN model predicts the selected 111 stocks' prices, they are compared with the SSE 50 Index and CSI 500 Index data results. The specific results are unfolded in Figure 13.

From Figure 13, the DL NN model-predicted stock portfolio, SSE 50 Index, and CSI 500 Index all show a fluctuating trend. Compared with the trend of the other two stocks, the DL NN model-predicted stock portfolio shows good adaptability. It can quickly adjust the strategy when the stock trend is low, maintaining corresponding stability. Thus, it is possible to seize the favorable market prospect, seize investment, and strengthen stocks' continuous recovery and growth.

**Figure 14:**
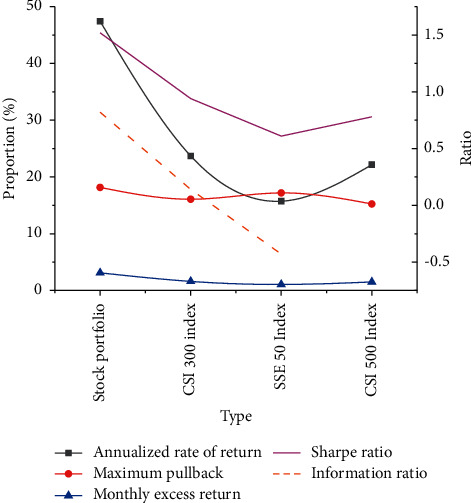
Comparison of relevant returns between proposed DL NN-predicted stock portfolio and other stocks.

Additionally, the DL NN model-predicted stock portfolio is compared with CSI 300 Index from annualized PR, SR, maximum pullback, monthly excess PR, and information ratio. The specific results are painted in Figure 14.

From Figure 14, the annualized PR, SR, maximum pullback, monthly excess PR, and information ratio are 47.44%, 1.52, 18.15%, 3.11%, and 0.82, respectively. By comparison, the annualized PR, SR, the maximum pullback, monthly excess PR, and information ratio of the CSI 300 Index are 23.69%, 0.94, 16.08%, 1.6%, and 0.14, respectively. Lastly, those indexes of the SSE 50 Index are 15.73%, 0.61, 17.19%, 1.05%, and −0.43, respectively. On the other hand, the annualized PR, SR, the maximum pullback, and the monthly excess PR of the SSE 50 Index are 22.16%, 0.78, 15.24%, and 1.5%, respectively. Accordingly, the stock portfolio prediction based on the DL NN model shows the best results in all dimensions. The performance of the proposed DL NN-predicted stock portfolio continues to be better than the large market stock index to a great extent.

## 4. Conclusion

With the continuous improvement of the social and economic level and the rapid financial market development, more people choose to invest in the stock market to achieve excess returns. However, the investment environment shows a certain complexity. This study builds a stock portfolio investment strategy based on the DL NN model and draws the following conclusions. (1) 111 stable stocks are selected from the CSI 300 Index as the research samples. The data results reveal that during the four years from January 1, 2018, to December 31, 2021, the stock's closing price is relatively stable, showing a trend of up and down fluctuations around the horizontal axis. The positive and negative returns are also balanced, roughly between −5% and 5%. There is a phenomenon of fluctuation aggregation to a certain extent. (2) The relevant data of 111 stocks are imported into the DL NN model to obtain the prediction results of stock closing prices. It finds that model *c* performs the best. With the increase in the number of input variables, the NN prediction result is also improved. (3) The MSE and MAE of the closing price prediction results and yield prediction results are analyzed. The findings imply that the MSE and MAE show a downward trend with the continuous increase in model input, and the error is getting smaller and smaller. (4) The relevant returns of the constructed stock portfolio are compared with other stocks. As a result, annualized return, Sharpe ratio, maximum drawdown, monthly excess return, and final information ratio of the proposed stock portfolio based on the DL NN model present the best results. However, due to the many uncertainties in the stock market, it is still challenging to achieve accurate forecasts.

Still, the article has some limitations in data acquisition, resulting in some deviations in the test of relevant data. The construction of a stock portfolio prediction based on DL NN has not been discussed regarding economic cost investment. The follow-up study will introduce the benefit evaluation according to the specific situation. It aims to provide a theoretical optimization basis and direction for investors and other investment entities.

## Figures and Tables

**Table 1:** Parameter setting of DL NN model.

Type	Value
Network node	75% of total points
Initial weight	−0.5∼0.5
Minimum training rate	0.9
Dynamic parameter	0.6∼0.8
Allowable error	0.001∼0.00001
Number of iterations	1000
Sigmoid parameters	0.9∼1

## Data Availability

The data used to support the findings of this study are included within the article.
